# Isolated CNS Whipple disease with normal brain MRI and false-positive CSF 14-3-3 protein: a case report and review of the literature

**DOI:** 10.1002/brb3.97

**Published:** 2012-10-01

**Authors:** Victor W Sung, Michael J Lyerly, Kenneth B Fallon, Khurram Bashir

**Affiliations:** 1 Department of Neurology, University of Alabama at BirminghamBirmingham, Alabama; 2 Department of Pathology, University of Alabama at BirminghamBirmingham, Alabama

**Keywords:** Infectious disease, MRI, neuroimaging, neurology, Whipple disease

## Abstract

Whipple disease (WD) is usually a systemic infectious disease that can have central nervous system (CNS) involvement. WD confined to the CNS is extremely rare and difficult to diagnose, but can be fatal if not treated in a timely fashion. We present the case of a 42-year-old man with a subacute dementia accompanied by a movement disorder consisting of progressive supranuclear gaze palsy, myoclonus, and ataxia. Our patient lacked the typical magnetic resonance imaging (MRI) findings reported with isolated CNS WD and had a false-positive cerebrospinal fluid (CSF) 14-3-3 protein. The patient expired, and definitive diagnosis of isolated CNS WD was made by autopsy with characteristic macrophage accumulations found in the brain but not in the gastrointestinal tract. We examine the literature on isolated CNS WD and discuss how these previously unreported findings make a rare diagnosis even more challenging. The reported patient is the first in the literature with tissue diagnosis of isolated CNS WD in the setting of normal brain MRI and positive CSF 14-3-3 protein. Isolated CNS WD should be added to the list of considerations for a false-positive CSF 14-3-3 protein. Even in the absence of typical MRI lesions, a patient with subacute progressive dementia, supranuclear gaze palsy, and other various neurologic abnormalities should have the diagnosis of isolated CNS WD considered.

## Background

Whipple disease (WD) is a rare infectious disease caused by the Gram-positive bacillus *Tropheryma whipplei* that most commonly affects the gastrointestinal system with symptoms of chronic diarrhea and malabsorption but can also affect many other organ systems (Dobbins and Ruffin 1967; La Scola et al. 2001; Fenollar et al. 2007). The central nervous system (CNS) is involved in up to 40% of cases and can have widely variable neurologic manifestations (Sieracki et al. 1960; Halperin et al. 1982; Fleming et al. 1988; Louis et al. 1996; Verhagen et al. 1996, 1997; Durand et al. 1997; Anderson 2000; La Scola et al. 2001; Gerard et al. 2002; Scheld 2003; Marth 2009). In rare instances, the neurologic symptoms can occur without the gastrointestinal symptoms, and this has been reported in 34 cases (Verhagen et al. 1996; Gerard et al. 2002; de Andrade et al. 2007; Benito-Leon et al. 2007, 2008; Marth 2009; Black et al. 2010). However, in even more rare cases (21 reported), the infectious disease is confined to the CNS only, with no systemic manifestations, in what is known as isolated CNS WD (Panegyres et al. 2006; Marth 2009; Black et al. 2010). The absence of gastrointestinal and systemic symptoms makes isolated CNS WD difficult to diagnose, and WD is invariably fatal without timely treatment (Keinath et al. 1985; Feurle and Marth 1994; Marth 2001; Panegyres et al. 2006; Schneider et al. 2008). Diagnosis is usually made when abnormal magnetic resonance imaging (MRI) and characteristic neurologic exam findings lead to confirmatory laboratory testing, but this depends on both the ability to detect the exam findings and the presence of the MRI abnormalities. We discuss this diagnostic challenge, particularly when pathognomonic exam findings are not detected and MRI abnormalities are not present, in the context of a clinical case report and review of the literature.

## Case Report

### History

A 42-year-old Caucasian man was admitted to the inpatient neurology service at our hospital for evaluation of progressive neurologic deterioration. Approximately 18 months prior to admission, the patient started to have difficulty sleeping characterized by frequent nighttime awakenings and daytime somnolence. After seeing a sleep specialist, he was diagnosed with obstructive sleep apnea and periodic limb movements of sleep. Despite treatment with continuous positive airway pressure (CPAP) and sleep aids, he continued to have poor sleep and his abnormal movements worsened. Six months prior to admission, the patient began having spells that were characterized by video electroencephalogram (EEG) monitoring as frontal lobe seizures and was started on topiramate.

Over the ensuing months leading up to admission, he experienced a cognitive decline with impaired short-term memory, disinhibition, and visual hallucinations. His gait gradually became unstable with a stooped posture which led to frequent falls. At the time of admission, he was no longer able to stand without assistance. He had also developed multifocal jerking movements of all of his extremities as well as dysphagia with a 100 pound weight loss over 6 months despite absence of abdominal pain or diarrhea.

### Physical examination

His initial examination revealed a chronically ill appearing male who required assistance for all of his activities of daily living. He was afebrile with an unremarkable general physical examination. He was fully oriented; however, his speech was extremely dysarthric. There was no evidence of aphasia. His pupils were equal but minimally reactive. He had a complete vertical gaze palsy and partial horizontal gaze impairment to both smooth pursuit and saccades. These could be overcome by oculovestibular maneuvers. Visual fields were intact to confrontation. He had facial diplegia and myoclonus of the face was noted, although it was not labeled as oculomasticatory myorhythmia (OM) because eye movements were not specifically examined for this. He had intact facial sensation. On motor examination, he had symmetric diffuse 4/5 strength except for 3/5 strength in the bilateral iliopsoas muscles. He had a head drop which he was unable to voluntarily overcome. His tone was mildly increased throughout the bilateral upper and lower extremities, and axial rigidity was present as well. There was diffuse atrophy, particularly of the hand intrinsic muscles. Myoclonus was also seen in all extremities. His sensory examination was normal to all modalities. Reflexes were normal in the upper extremities but hyperactive in the lower extremities. Dysmetria was present in the upper extremities, and he had truncal ataxia when he sat up in bed. Upon standing, he had extreme stooping of posture, and his gait was slow and unsteady, requiring constant assistance.

### Investigations

**Figure 1:**
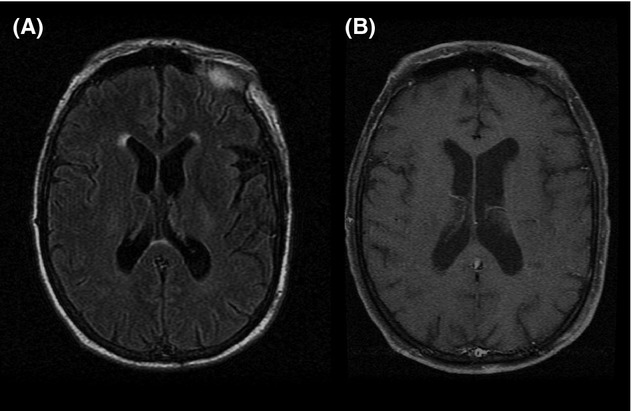
MRI images obtained on admission. (A) Axial FLAIR image that is unremarkable, without any significant hyperintensities. (B) Axial T1-weighted postcontrast image that is unremarkable, showing no abnormal areas of enhancement.

MRI of the brain performed on day 1 of admission was unremarkable except for mild diffuse atrophy, and specifically, there were no abnormal hyperintense or contrast-enhancing lesions (Fig. 1). Two EEG's were performed, both of which showed mild–moderate diffuse slowing. Routine cerebrospinal fluid (CSF) studies were normal except for a slightly elevated protein. Due to the rapidly progressive dementia, CSF 14-3-3 protein was sent. Needle electromyography (EMG) demonstrated acute and chronic denervation in the upper and lower extremities. Laboratory studies for paraneoplastic antibodies were negative, and CT of the chest, abdomen, and pelvis was unrevealing for a primary neoplasm. Neuropsychological testing revealed a dementia with multiple domains affected, most prominently in executive function and language processing.

### Clinical course

The patient was given trials of carbidopa/levodopa, clonazepam, and ropinirole with only minimal improvement in his jerking. Over the 2-week hospital stay, he became progressively weak to the point where he was bedbound. His dysphagia also worsened to the point that he required placement of a gastrostomy tube. His CSF 14-3-3 returned as “elevated compared with the normal control,” but at the time this patient was seen, the National Prion Disease Pathology Surveillance Center was not reporting tau levels so a quantification was not possible. As the patient lacked the other supportive features for Creutzfeldt–Jakob disease (CJD) such as characteristic MRI and EEG, we considered the 14-3-3 result to be false positive for CJD, and the patient was discharged to a skilled nursing facility with a presumed degenerative disorder such as a Parkinson-plus syndrome but without a definitive diagnosis. The patient continued to have progressive worsening of his condition, and he expired 2 months following discharge.

### Autopsy and diagnosis confirmation

**Figure 2:**
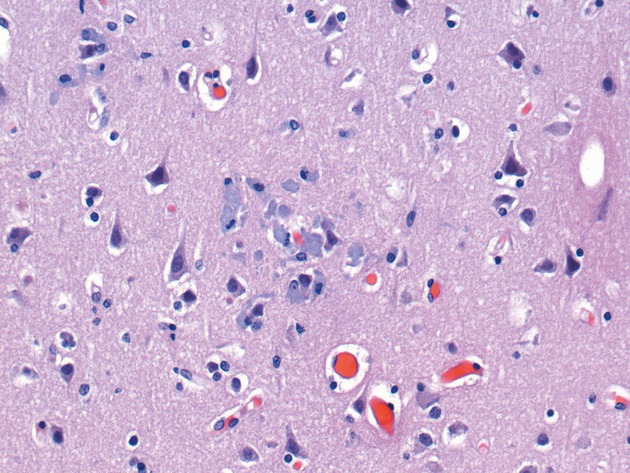
Macrophages having distended, pale basophilic cytoplasm in cerebral cortex (×200, hematoxylin and eosin).

**Figure 3:**
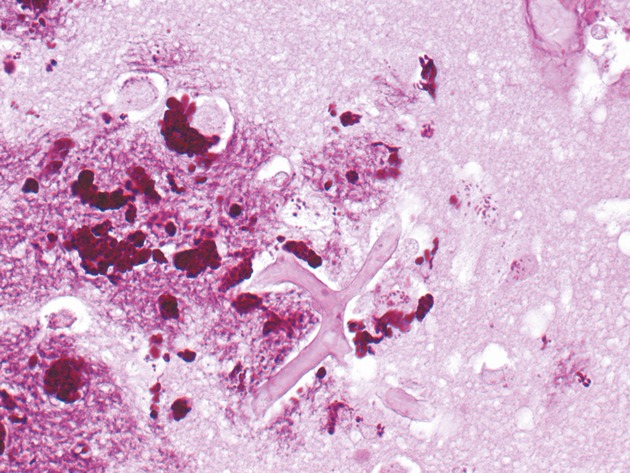
Numerous diastase-resistant intracytoplasmic and extracellular organisms of *Tropheryma whipplei* near perivascular branch points in cerebral cortex (×400, periodic acid-Schiff [PAS] with diastase).

**Figure 4:**
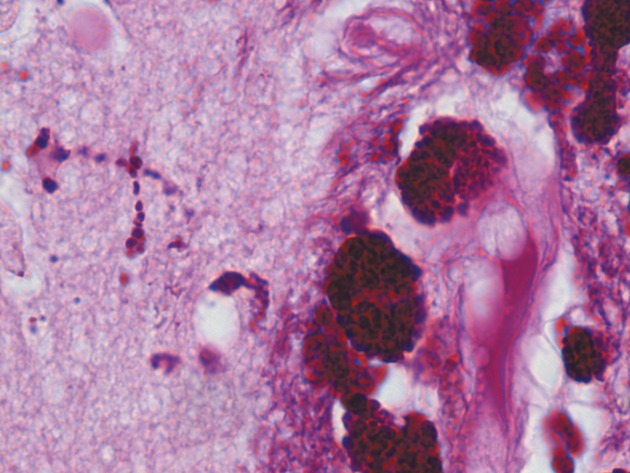
Free *Tropheryma whipplei* organisms in neuropil (left) adjacent to a perivascular region where numerous intracellular *T*. *whipplei* organisms are present within macrophages (×1000, PAS with diastase).

An autopsy was requested to confirm his diagnosis. Microscopic analysis of his brain tissue revealed perivascular and intraparenchymal accumulations of basophilic macrophages scattered through the cerebral cortex, basal ganglia, and brainstem (see Fig. 2). The lipid-filled cytoplasm of the macrophages contained sickle-shaped inclusions that were intensely positive with periodic acid-Schiff (PAS), Gram, and gomori-methenamine (GMS) stains, showing that the inclusions consisted of Gram-positive bacteria (see Fig. 3). These bacteria were also present extracellularly (see Fig. 4). Further analysis of tissue from other organs, including multiple samples from the gastrointestinal tract, was performed, and no other accumulations of macrophages or Gram-positive bacteria were found. A tissue sample was sent to the National Prion Disease Pathology Surveillance Center and was negative for prion protein. His diagnosis was changed to isolated CNS WD based on the neuropathologic findings.

## Discussion and Literature Review

Our case illustrates many important points in the diagnosis and treatment of isolated CNS WD. First, if this rare and challenging diagnosis is made late or not at all, then it may lead to death. Any CNS involvement in WD carries a poor prognosis, with 25% of patients dying and another 25% having major neurologic sequelae within 4 years of diagnosis (Schnider et al. 1996). Despite this, WD is ultimately still an infectious bacterial disease that can respond to early antibiotic treatment, which requires early diagnosis (Keinath et al. 1985; Feurle and Marth 1994; Marth 2001; Schneider et al. 2008). All cases of isolated CNS WD are challenging because of the lack of gastrointestinal or other systemic findings that are seen in WD with CNS involvement. Many reported cases of CNS WD had early predominant GI features and therefore had a known diagnosis of WD prior to development of neurologic symptoms.

Our case of isolated CNS WD presented as a progressive disorder with dementia, supranuclear gaze palsy, myoclonus, and gait disorder with ataxia. Phenomenologically, the most commonly described movement disorder seen in CNS WD is OM, and it has even been suggested to be pathognomonic for CNS WD (Schwartz et al. 1986; Louis et al. 1996; Revilla et al. 2008). OM is characterized by continuous horizontal movements of the eyes, converging in and then back out to primary position with very small amplitude and at a frequency of roughly 1 Hz (Fahn et al. 2011). The images shown by Revilla et al. (2008) and the video in the previously cited textbook are extraordinarily helpful to recognize OM, but also show how subtle it is to recognize despite the facial movements usually occurring at about the same frequency. As OM frequently occurs with a vertical supranuclear gaze palsy (Fahn et al. 2011), which our patient was documented to have, we may have missed the presence of OM due to its subtlety or it may have been completely absent. Another case of isolated CNS WD has been reported with absence of OM in the setting of facial paralysis (Verhagen et al. 1996), and facial paresis in CNS WD has been reported on numerous occasions (Hausser-Hauw et al. 1988; Simpson et al. 1995; Coene et al. 1996; Louis et al. 1996; Akar et al. 2002). Our patient also had ataxia and myoclonus, which have been described extensively in CNS WD (Halperin et al. 1982; Louis et al. 1996; Verhagen et al. 1997; Anderson 2000; Scheld 2003; Matthews et al. 2005; Panegyres et al. 2006).

In our case, the neuropsychologist felt that the pattern of dementia was consistent with what is seen in progressive supranuclear palsy (PSP), but the overall clinical progression was more rapid than what is typically seen in PSP. Generally, progression of CNS symptoms in isolated CNS WD is subacute and progressive, as was seen in our patient. However, occasionally progression can occur in a relapsing–remitting pattern (Benito-Leon et al. 2007) or an acute stroke-like pattern (Peters et al. 2002; Famularo et al. 2005). Other reported neurologic signs and symptoms in CNS WD span nearly the entire neurologic spectrum, including seizures, hemiplegia, headaches, cranial neuropathies, weakness, neglect, increased or decreased reflexes, and sensory loss (Panegyres et al. 2006). Therefore, presentation with any of the above findings, particularly supranuclear gaze palsy (even in the presence of other features suggestive of PSP), should prompt a closer evaluation for OM and consideration of CNS WD as an alternative diagnosis.

Diagnosis of our patient's condition was further confounded by the lack of MRI lesions typically seen in isolated CNS WD and the presence of a false-positive CSF 14-3-3 protein. To our knowledge, neither of these has been reported previously with isolated CNS WD. Although isolated CNS WD is rare, there are sufficient cases for proposal of two distinct imaging presentations. Panegyres et al. (2006) propose that the two recognizable imaging presentations in isolated CNS WD are (A) multiple enhancing lesions on CT or MRI correlating with various neurologic signs/symptoms and (B) solitary mass lesions on CT or MRI resulting in focal neurologic symptoms. In their review of cases in the literature, only one other case of suspected isolated CNS WD had no lesions on imaging (Louis et al. 1996), but they excluded this case due to the lack of confirmatory tissue or molecular pathology. Our review of that case reveals that the presentation was similar to our patient, with supranuclear gaze palsy and extrapyramidal symptoms. It has been reported previously that systemic WD with extension to the CNS typically has an imaging appearance consistent with a basal encephalitis and/or ependymitis (Grossman et al. 1981; Schnider et al. 1995) but can have normal imaging (Black et al. 2010). Our case and the above unconfirmed case suggest that in addition to systemic WD that extends to the CNS, isolated CNS WD can also have normal imaging. Therefore, when there is reasonable clinical suspicion for CNS WD, it should not be ruled out simply due to an absence of MRI lesions.

Our patient had a positive CSF 14-3-3 protein but did not have CJD. The CSF 14-3-3 protein has a high sensitivity and specificity for CJD, such that in cases of rapidly progressive dementia, it is a recommended test by the American Academy of Neurology for confirming or excluding the diagnosis of CJD (Knopman et al. 2001). However, false-positive CSF 14-3-3 protein has been described in many cases of rapidly progressive dementia, with diagnoses as varied as Alzheimer's disease, multiple sclerosis, stroke, glioma, CNS vasculitis, paraneoplastic disorders, and Down syndrome (Saiz et al. 1998, 1999; Kenney et al. 2000; Lemstra et al. 2000; Zerr et al. 2000; Burkhard et al. 2001; Peoc'h et al. 2001). In our review of the literature, no previous report of CNS WD in any form has had documented positive CSF 14-3-3 protein. As previously discussed, CJD was not a strong consideration in this case given the absence of typical EEG and MRI findings, but our case is useful in adding CNS WD to the list of diagnoses that should be considered when CSF 14-3-3 is positive, but the clinical picture does not fit with CJD.

For the reasons outlined above, our case illustrates how difficult the diagnosis of isolated CNS WD can be to make. However, it also bears describing what the proper diagnostic and therapeutic steps should be if the diagnosis of isolated CNS WD is properly made. In CNS WD with gastrointestinal symptoms, the initial diagnostic procedure should be upper endoscopy with small bowel biopsy looking for *T. whipplei* (Marth 2001, 2009); however, isolated CNS WD by definition will have a negative biopsy, as was confirmed in our case with postmortem GI tract evaluation negative for *T. whipplei*. For isolated CNS WD, it has been suggested that a combination of neuroimaging and *T. whipplei* polymerase chain reaction (PCR) evaluation on the CSF be used as the standard for diagnosis (Panegyres et al. 2006). Given our report here of isolated CNS WD with normal MRI, we would propose the use of CSF PCR for *T. whipplei* as the primary confirmatory diagnostic test for isolated CNS WD. It has been suggested that CSF oligoclonal bands may be useful in monitoring response to treatment (Panegyres et al. 2006).

The current treatment recommendation for WD is intravenous ceftriaxone, 2 g every 12 h for 2 weeks followed by oral double strength trimethoprim–sulfamethoxazole twice daily for 1–2 years (Feurle and Marth 1994; Marth 2001, 2009). In general WD, the gastrointestinal symptoms respond first, but response of neurologic symptoms, particularly in CNS WD and isolated CNS WD, may require weeks to months for a response, with some patients experiencing relapse and/or death despite treatment (Feurle and Marth 1994; Famularo et al. 2005; Panegyres et al. 2006).

In summary, a high index of suspicion for isolated CNS WD should be maintained for patients presenting with rapidly progressive cognitive decline with supranuclear gaze palsy or other neurologic signs and negative workup for more common etiologies. This suspicion should remain high even in the absence of gastrointestinal symptoms and unexplained MRI lesions, and in the presence of a positive CSF 14-3-3 protein. Suspicion for any form of CNS WD should prompt careful evaluation for oculomasticatory myorhythmia and CSF PCR for *T. whipplei*. Timely diagnosis and treatment of isolated CNS WD (and WD in general) is critical to prevent a potentially fatal outcome.
